# Cross species selection scans identify components of C_4_ photosynthesis in the grasses

**DOI:** 10.1093/jxb/erw256

**Published:** 2016-07-19

**Authors:** Pu Huang, Anthony J. Studer, James C. Schnable, Elizabeth A. Kellogg, Thomas P. Brutnell

**Affiliations:** 1 Donald Danforth Plant Science Center, 975 N Warson Rd, St Louis, MO 63132, USA; 2 Department of Crop Sciences, University of Illinois Urbana-Champaign, Urbana, IL 61801, USA; 3 Department of Agronomy and Horticulture, University of Nebraska-Lincoln, Lincoln, NE 68588, USA

**Keywords:** Adaptation, C_4_ photosynthesis, cross-species selection scans, gene discovery, grasses, parallel evolution.

## Abstract

C_4_ photosynthesis is perhaps one of the best examples of convergent adaptive evolution with over 25 independent origins in the grasses (Poaceae) alone. The availability of high quality grass genome sequences presents new opportunities to explore the mechanisms underlying this complex trait using evolutionary biology-based approaches. In this study, we performed genome-wide cross-species selection scans in C_4_ lineages to facilitate discovery of C_4_ genes. The study was enabled by the well conserved collinearity of grass genomes and the recently sequenced genome of a C_3_ panicoid grass, *Dichanthelium oligosanthes*. This method, in contrast to previous studies, does not rely on any *a priori* knowledge of the genes that contribute to biochemical or anatomical innovations associated with C_4_ photosynthesis. We identified a list of 88 candidate genes that include both known and potentially novel components of the C_4_ pathway. This set includes the carbon shuttle enzymes pyruvate, phosphate dikinase, phosphoenolpyruvate carboxylase and NADP malic enzyme as well as several predicted transporter proteins that likely play an essential role in promoting the flux of metabolites between the bundle sheath and mesophyll cells. Importantly, this approach demonstrates the application of fundamental molecular evolution principles to dissect the genetic basis of a complex photosynthetic adaptation in plants. Furthermore, we demonstrate how the output of the selection scans can be combined with expression data to provide additional power to prioritize candidate gene lists and suggest novel opportunities for pathway engineering.

## Highlight

A new genome wide scan method using signals of positive selection identifies C_4_ candidate genes in the grasses independent of *a priori* knowledge of C_4_ biochemistry.

## Introduction

C_4_ photosynthesis evolved multiple times coincident with a steep decline in global CO_2_ levels approximately 30–40 mya (Giussani *et al.*, 2001; Sage, 2004; Vicentini *et al.*, 2008; Edwards and Smith, 2010; Sage *et al.*, 2011, 2012). This correlation suggests that C_4_ adaptively evolved as a mechanism to concentrate carbon in the vicinity of ribulose-1,5-bisphosphate carboxylase/oxygenase (rubisco), thus significantly reducing energetic losses associated with photorespiration (Sage, 2004; Sage *et al.*, 2011, 2012). The majority of C_4_ plants utilize two dimorphic cell types to fix CO_2_. Bundle sheath (BS) cells perform most of the reactions required for the Calvin cycle and some cyclic electron transport while the surrounding mesophyll (M) cells serve as the initial site of carbon capture and perform linear electron transport to drive the production of NADPH and ATP. BS and M cells form a wreath-like structure surrounding vasculature tissues known as Kranz anatomy. This is most often associated with C_4_ photosynthesis (Brown, 1975; Giussani *et al.*, 2001; Sage *et al.*, 2011, 2012). This morphological adaptation and associated division of biochemical activities serves to pump C_4_ acids into the BS that are later decarboxylated in the BS plastid where most Calvin cycle enzymes are localized. These innovations have resulted in some of the most productive plants on the planet, accounting for an estimated 25% of global primary production, despite including only 3% of all angiosperm species (Still *et al.*, 2003). Traditionally, C_4_ species have been classified into three major subtypes based on the primary decarboxylating enzyme present in the BS (Sage, 2004; Furbank, 2011; Sage *et al.*, 2011): NADP malic enzyme (NADP-ME), NAD malic enzyme (NAD-ME) and phosphoenolpyruvate carboxykinase (PCK).

The evolution of the C_4_ carbon pump involves a number of dramatic changes: increased vein density, increased photosynthetic capacity of the BS cells, repositioning of organelles, changes in photosynthetic membranes, and the redistribution of enzymes into subcellular compartments. In many cases, genes encoding proteins that perform other functions in C_3_ plants have been co-opted into new roles in C_4_ photosynthesis (Sage *et al.*, 2011, 2012). Molecular approaches to dissect the regulatory networks guiding C_4_ differentiation have focused on profiling or co-expression studies that often yield hundreds to thousands of candidate genes (Li *et al.*, 2010; Chang *et al.*, 2012; John *et al.*, 2014; Wang *et al.*, 2014*a*; Huang and Brutnell, 2016), with little evidence for prioritization. Reverse genetic screens have largely been limited to known components such as carbon shuttle enzymes (Bailey *et al.*, 2000; Cousins *et al.*, 2006; Studer *et al.*, 2014) and have not yielded insights into networks regulating the differentiation process. Comparative studies of molecular evolution, on the other hand have shown core C_4_ genes such as phosphoenolpyruvate carboxylase (PEPC) (Christin *et al.*, 2007), NADP-ME (Christin *et al.*, 2010) and PCK (Christin *et al.*, 2009) to be adaptively evolving in C_4_ clades. However, like reverse genetic screens, these studies relied on *a priori* information on the biochemistry of the C_4_ carbon shuttle pathway to first identify gene candidates.

In this report we describe a novel method to use signals of adaptive evolution to identify candidate genes required for C_4_. The method conducts an automated genome-wide scan and does not rely on *a priori* information to define candidates. Rather, putative C_4_ genes are identified based strictly on the ratio of rates of nucleotide substitutions. We focus our study on the grasses (Poaceae), as C_4_ has originated in grasses at least 25 times and they include some of the most ecologically successful C_4_ species (Giussani *et al.*, 2001; Sage *et al.*, 2011; Grass Phylogeny Working Group II, 2012). We identify 88 genes that show potential adaptive evolution in C_4_ lineages. These genes include both known components of the C_4_ pathway and several suspected and novel components. When coupled with expression profiling, this approach provides a powerful tool for gene discovery and potentially for engineering alternative forms of C_4_ photosynthesis.

## Materials and methods

### Obtaining syntenic orthologs and quality control

Reference primary coding DNA sequences (CDSs) of rice, *Brachypodium distachyon*, *Setaria italica*, sorghum and maize were downloaded from Phytozome 10 (http://phytozome.jgi.doe.gov). The CDSs of *Dichanthelium oligosanthes* were obtained from CoGe (http://genomevolution.org, genome ID no. 20291) (A. J. Studer, J. C. Schnable, S. Weissmann *et al.*, unpublished data). Lists of known syntenic orthologs were obtained from (Schnable *et al.*, 2012), and *S. italica* syntenic orthologs were identified using the same method as described in (Schnable *et al.*, 2012). Ortholog groups that were duplicated in the maize whole genome duplication event (Schnable *et al.*, 2009) were merged, and BLASTN (Camacho *et al.*, 2009) was used to identify the closest *D. oligosanthes* homolog to the *S. italica* ortholog. This yielded 16 943 ortholog groups. We then considered four patterns of gene relationship: (i) one ortholog in all six species (8143); (ii) two orthologs in maize (homeologs) and one ortholog in the other five species (3262); (iii) rice ortholog missing and one ortholog in the other five species (1029), and (iv) *B. distachyon* ortholog missing and one ortholog in the other five species (604). Blast hits without gene annotation were considered missing. These patterns were specifically considered because C_4_ branches can be unambiguously assigned. Collectively these occasions accounted for about 77% (13 038 out of 16 934) of ortholog groups. Codon-based alignment was performed using ProGraphMSA (Szalkowski, 2012), and the resulting alignments were trimmed using Gblocks (Castresana, 2000) and short alignments (less than 30% coverage) discarded. A maximum likelihood (ML) phylogenetic tree was constructed using RaxML (Stamatakis, 2014) using all sites and MEGA-CC (Kumar *et al.*, 2012) using only the third position of codons. The GTR+gamma+I mutation model was used in both analyses. Resulting trees were then compared with the species phylogeny and tested for topological congruence using qdist (Mailund and Pedersen, 2004). Failing both phylogenetic congruence tests resulted in exclusion from further analysis. Finally 6784 ortholog groups were obtained.

### Test for potential selection and identification of candidate genes

**Fig. 1.**
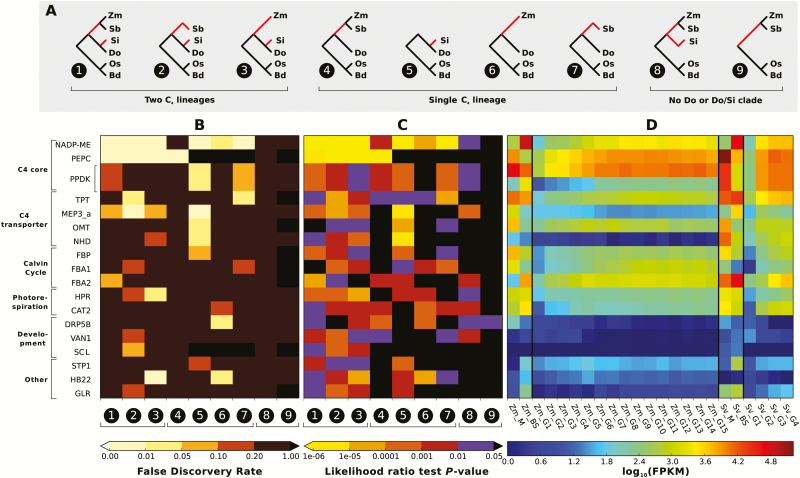
Phylogenies used for selection scan, statistical significance and tissue-specific expression data for top 18 candidate C_4_ ortholog groups. (A) Nine phylogenetic conditions used for selection scan. Red branches are branches where the C_3_ to C_4_ transitions are inferred to have occurred (C_4_ branches). Zm: maize; Sb: sorghum; Si: *S. italica*; Do: *D. oligosanthes*; Os: rice; Bd: *B. distachyon*. (B) False discovery rates from selection scan. Each column represents tests under the same phylogenetic condition corresponding to (A), and each row (or two rows in the case of maize, which has two homeologs) represent one ortholog group. Lighter color indicates higher significance. Ortholog groups are grouped according to their functional relevance to C_4_, specified on the left. (C) *P*-values of likelihood ratio tests from selection scan. These are single test statistics and not multi-tests corrected. (D) Tissue specific expression profile of corresponding ortholog groups in maize and *Setaria*, shown on log scale. Zm_M/BS: maize mesophyll/bundle sheath; Zm_G1-15: maize leaf gradient. Sv_M/BS: *Setaria viridis* mesophyll/bundle sheath; Sv_G1-4: *S. viridis* leaf gradient. The BS/M original data are downloaded from John *et al.* (2014) and were originally generated by John *et al.* (2014) and Chang *et al.* (2012). The maize leaf gradient data are obtained from Wang *et al.* (2014*a*). The *S. viridis* gradient data are obtained from (A. J. Studer, J. C. Schnable, S. Weissmann *et al.*, unpublished data).

The branch model of PAML 4.2 (Yang, 2007) was used to calculate likelihoods of the data given the null hypothesis (H_0_) assuming all branches shared the same ratio of *d*_N_/*d*_S_, and the alternative hypothesis (H_a_) assuming C_4_ branches had a *d*_N_/*d*_S_ ratio independent from all other branches (Fig. 1A). A likelihood ratio test was used to evaluate the significance of H_a_ over H_0_ (Yang, 2007). The full phylogeny with all six species (condition 1) theoretically requires an ortholog group to be under positive selection in all three C_4_ species. In order to account for possible selection that only occurred in specific subsets of C_4_ lineages, additional tests under six conditions with one or two C_4_ lineages manually removed were considered. These conditions include maize removed (condition 2), sorghum removed (condition 3), *S. italica* removed (condition 4), the maize–sorghum clade removed (condition 5), sorghum and *S. italica* removed (condition 6), and maize and *S. italica* removed (condition 7). To determine the importance of the *Setaria*–*Dichanthelium* clade, two additional conditions were also considered in which *Dichanthelium* (condition 8) and the *Setaria*–*Dichanthelium* clade (condition 9) were removed manually (Fig. 1A). The testing topologies under these conditions were further modified in cases when maize duplication and rice/*Brachypodium* gene loss needed to be accounted for, and tests were not conducted if there were less than four taxa available (for final testing topologies see Supplementary Table S1 at *JXB* online).

A multi-test correction was performed under each phylogenetic condition to obtain the false discovery rate (FDR) using the R package fdrtools (Strimmer, 2008). Ortholog groups with FDR<0.2 in at least one test (indicating an elevated *d*_N_/*d*_S_ ratio in at least one C_4_ branch) were merged to generate a final candidate gene list, grouping by their putative relationship to C_4_. The cell-type and leaf gradient expression profile measured in fragments per kilobase of exon per million fragments mapped (FPKM) of these candidates in maize and *Setaria* were extracted from previous studies (Li *et al.*, 2010; Chang *et al.*, 2012; John *et al.*, 2014; Wang *et al.*, 2014*a*; A. J. Studer, J. C. Schnable, S. Weissmann *et al.*, unpublished data). A gene ontology enrichment analysis was also performed using the GO annotations of the closest *Arabidopsis thaliana* homolog using AgriGO (Du *et al.*, 2010) with the background as the non-redundant *A. thaliana* homolog of 6784 ortholog groups. Finally, we manually examined ten homolog groups that were putatively involved in C_4_ photosynthesis (Chang *et al.*, 2012; John *et al.*, 2014) but were filtered out from the automated workflow (Supplementary Table S2). Homologs were identified using BLASTN when syntenic orthologs are not available. Case-specific phylogenies were used to determine orthology and account for the complexities involved in these situations.

## Results

### An overview of the candidates and the automated workflow

**Fig. 2.**
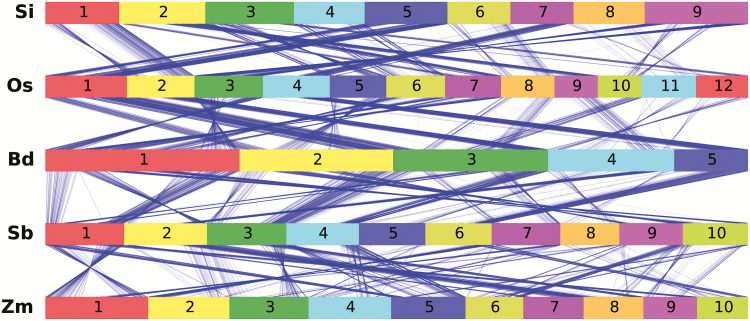
Gene synteny across five grass species (a random set of 1400 ortholog groups are shown). Si: *Setaria italica*; Os: *Oryza sativa* (rice), Bd: *Brachypodium distachyon*; Sb: *Sorghum bicolor* (sorghum); Zm: *Zea mays* (maize). Each colored segment represents one chromosome in one species, and the blue lines between species denote position of a pair of syntenic orthologs. Genome lengths of all species are normalized to be equal to each other.

Although phylogenetic relationships have been the subject of intense study in the grasses and several branches of C_3_ to C_4_ transitions defined (simplified as ‘C_4_ branches’ hereafter) (Christin *et al.*, 2007, 2009), gene duplication, loss and polyploidization confound attempts to streamline genome-wide scans (Wang *et al.*, 2009; Christin *et al.*, 2013). Thus, we have employed a set of orthologous relationships among five grass species based on syntenic conservation (Schnable *et al.*, 2012) (Fig. 2). These species are *Oryza sativa* (rice; Ouyang *et al.*, 2007), *Brachypodium distachyon* (Vogel *et al.*, 2010), *Setaria italica* (Bennetzen *et al.*, 2012), *Sorghum bicolor* (sorghum; Paterson *et al.*, 2009) and *Zea mays* (maize; Schnable *et al.*, 2009). Rice and *B. distachyon* employ C_3_ photosynthesis while the other three employ C_4_ photosynthesis. Among the three C_4_ species, maize and sorghum share a common origin of C_4_ while *S. italica* has evolved C_4_ photosynthesis independently (Giussani *et al.*, 2001; Edwards and Smith, 2010; Grass Phylogeny Working Group II, 2012). The relationships of genes present at syntenic locations in the genomes of multiple species strictly follow the phylogeny of the species themselves, meaning a uniform phylogeny can be applied to all genes for analysis. This makes it possible to conduct a cross-species genome scan in an automated fashion.

The most recent common ancestor of the C_3_ and C_4_ lineages included in the set of five grasses with complete assembled genomes represents at least ~50 million years of evolutionary divergence (Christin *et al.*, 2014). Accordingly, signals of positive selection may be obscured by many other randomly fixed changes along the long branches separating these grass lineages. Furthermore, while two independent origins of C_4_ (*Setaria* and maize–sorghum) are available, the C_3_ clades between these two C_4_ clades are not represented (Giussani *et al.*, 2001; Grass Phylogeny Working Group II, 2012). Thus the two independent origins of C_4_ are not distinguishable on the phylogeny of these five species. The recently published draft genome of *Dichanthelium oligosanthes*, a C_3_ panicoid grass, mitigates both issues described above (A. J. Studer, J. C. Schnable, S. Weissmann *et al.*, unpublished data). *D. oligosanthes* is more closely related to *Setaria* than it is to the maize–sorghum clade. Thus, inclusion of *D. oligosanthes* in our analysis greatly reduces divergence time between C_3_ and C_4_ lineages, and also phylogenetically separates *Setaria* from the maize–sorghum clade.

The genetic unit employed in this study is the pan-grass syntenic orthologous gene or ortholog group (Fig. 2). We define an ortholog group to be a set of genes that are syntenically orthologous across maize, sorghum, *Setaria*, rice, and *Brachypodium*, together with their putative *Dichanthelium* ortholog. A total of 13 038 ortholog groups were considered after grouping together syntenic homologs due to whole genome duplication in maize (Schnable *et al.*, 2009) and controlling for taxon coverage. Among them, 6784 ortholog groups passed both alignment quality and phylogenetic congruence tests, and were tested for potential positive selection using nonsynonymous to synonymous substitution rates ratio (*d*_N_/*d*_S_) based methods (see Materials and methods for details). Because elevated *d*_N_/*d*_S_ is a strong indication of positive selection or relaxed negative selection (Yang, 2007), we effectively conducted a cross-species selection scan. To account for the potential that different genes were co-opted in independent evolutionary origins of C_4_, an analysis was performed using the full phylogeny (condition 1), together with phylogenies with one (conditions 2–4) or two C_4_ species manually removed (conditions 5–7; Fig 1A). In total 88 ortholog groups were identified that show elevated *d*_N_/*d*_S_ in at least one C_4_ lineage after multi-test corrections (FDR<0.2, see Discussion), and 18 ortholog groups were prioritized based on their test significance and putative functions (Fig. 1; Supplementary Table S3). We also extracted expression data from published datasets (Chang *et al.*, 2012; John *et al.*, 2014; Wang *et al.*, 2014*a*) for further comparisons (Fig. 1).

### Core C_4_ genes

Of the five genes encoding the enzymes of the NADP-ME subtype carbon shuttle, three were among the resulting list of the automated workflow (Fig. 1). They include NADP-ME (Si000645m; for simplicity only the *Setaria* CDS is used unless otherwise necessary; for corresponding orthologs across all six species, see Supplementary Table S4), pyruvate, phosphate dikinase (PPDK; Si021174m) and PEPC (Si005789m). In both *Setaria* and maize these genes are highly expressed (fragments per kilobase of exon per million fragments mapped, FPKM>500) (Chang *et al.*, 2012; John *et al.*, 2014) in photosynthetic tissues, and thus are likely to be functional for photosynthesis (Fig. 1B, C, D). Another core C_4_ gene, NADP malate dehydrogenase (NADP-MDH; Si013632m) did not show evidence of adaptive evolution. A separate manual test for carbonic anhydrase (CA; Si003882m) was conducted because gene duplication and fusion resulted in its exclusion from the automated workflow (Studer *et al.*, 2014; A. J. Studer, J. C. Schnable, S. Weissmann *et al.*, unpublished data). Tests of CA based only on the putative photosynthetically active homologs (highly expressed homologs; Supplementary Table S2) failed to provide signals of adaptive evolution.

A proposed PCK pathway in maize (Wingler *et al.*, 1999) utilizes aspartate to shuttle carbon between M and BS. This pathway is maize specific, and thus was not included in the automated workflow. Manual examination of two syntenic orthologs of PCK, however, did reveal a signal of elevated *d*_N_/*d*_S_ in only one of the two (GRMZM2G001696; *P*=0.000000012; FDR for manual tests are not calculated because manual tests are case-specific; Supplementary Table S2). This ortholog shows high and biased expression in maize BS, consistent with a functional role in the PCK C_4_ pathway (Chang *et al.*, 2012). The two aspartate amino transferases (AspAT1 and AspAT2) did not show signals of adaptive evolution.

### Putative C_4_-related transporters

Of the six putative C_4_-related transport proteins (Kinoshita *et al.*, 2011; Furumoto *et al.*, 2011; Chang *et al.*, 2012; John *et al.*, 2014) that were included in the automated workflow, four were identified as targets of potential adaptive evolution (Fig. 1B, C; Supplementary Table S3). They include a dicarboxylate translocator (OMT, Si024403m), a putative pyruvate transporter (MEP3_a, Si024315m), an H^+^/Na^+^ antiporter relating to pyruvate transportation (NHD, Si029362m) and a triose-phosphate transporter (TPT; Si001693m). Another dicarboxylate translocator (DCT2, Si013503m) showed significance in a few single tests, but failed the corresponding multi-test corrections. Manual examinations of the other six ortholog groups, which were not included in the automated workflow due to our inability to unambiguously define orthology relationships, showed single test level significance in a dicarboxylate transporter (DCT4, Si035016m), a putative pyruvate transporter (sodium bile acid symporter BASS2, Si001591m) and a phosphoenolpyruvate/phosphate translocator (PPT1, Si013874m) (Supplementary Table S2). Tests for MEP3_c (Si005376m) were not conducted because a corresponding *Dichanthelium* homolog was not found. Among the three ortholog groups that did not show any signal of positive selection (MEP3_b, Si000451m; DCT1, Si029415m; PPT2, Si005351m), two showed low levels of expression in leaf tissue of *Setaria* and maize. In contrast, the ortholog groups that appear to have similar functions and show potential evidence of selection were all highly expressed in at least one C_4_ species (Supplementary Tables S2 and S3).

**Fig. 3.**
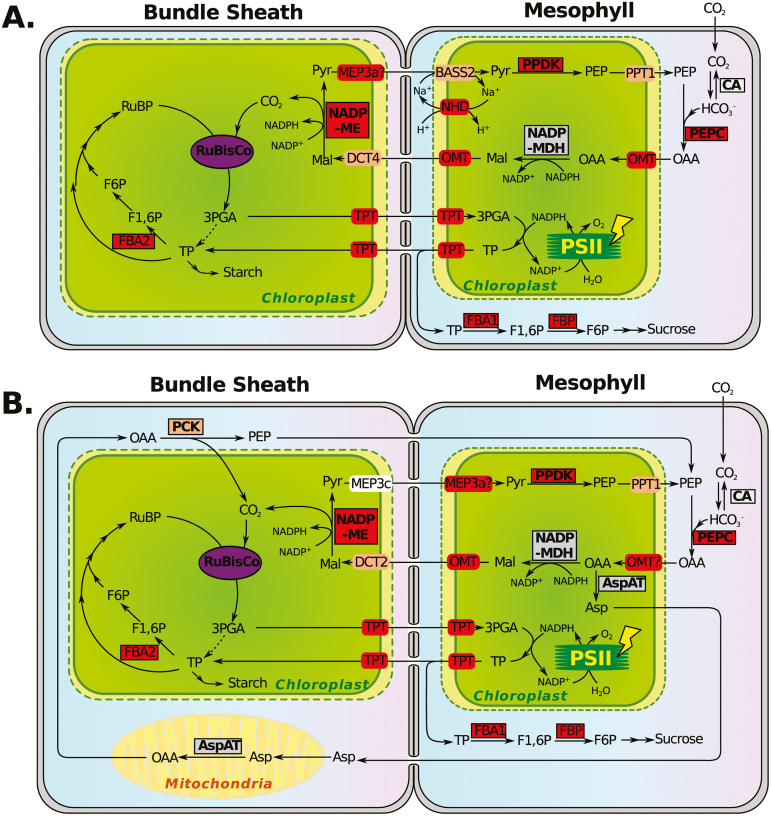
Hypothesized metabolite flow in (A) *Setaria italica/viridis* and (B) maize. Enzymes are enclosed in rectangles, and transporters are located on plastid membranes. The enzyme/transporter names correspond to those listed in Supplementary Tables S3 and S4. Enzymes and transporters colored in red show significant signal of positive selection (FDR<0.2) in at least one C_4_ lineage by the automated workflow. Those colored in orange are significant only at the single test level (*P*<0.01) in the automated workflow or manually, those colored in grey show no signal of positive selection in any test performed, and MEP3_c colored in white means meaningful tests could not be performed. 3PGA: 3-phosphoglycerate; Asp: aspartate; F1,6P: fructose-1,6-bisphosphate; F6P: fructose-6-phosphate; Mal: malate; OAA: oxaloacetate; PEP: phosphoenolpyruvate; Pyr: pyruvate; RuBP: ribulose bisphosphate; TP: triose phosphate.

Combining our results with bundle sheath/mesophyll (BS/M) expression profiles, proteomics and models of metabolite flow from previous studies (Aoki *et al.*, 1992; Majeran and van Wijk, 2009; Kinoshita *et al.*, 2011; Furumoto *et al.*, 2011; Chang *et al.*, 2012; John *et al.*, 2014), we generated a hypothesized overview of the adaptively evolving C_4_-related enzymes and transporters in maize and *Setaria* (Fig. 3). Although some uncertainties remain, an important observation for the C_4_ transporters is that the homolog groups showing potential evidence of selection collectively cover most plastidial transport roles needed for the NADP-ME subtype of C_4_ based on their putative function (Fig. 3). These results suggest that plastid membrane transporters in general are key components of C_4_ adaptive evolution, in addition to core C_4_ enzymes. Unlike the core C_4_ genes in which the same ortholog groups have been recruited in parallel, *Setaria* and the maize–sorghum lineages sometimes adopt transporters from different ortholog groups to achieve similar functions. This result reflects the great flexibility in biochemistry of the parallel C_4_ origins.

### Calvin–Benson–Basham cycle and photorespiration-related genes

Both the Calvin–Benson–Basham (CBB) cycle and photorespiration are processes that are predominantly BS-localized in C_4_ photosynthesis. As shown in Fig. 1 and Supplementary Table S3, two fructose-1,6-bisphosphate aldolases (FBAs) and one fructose-1,6-bisphosphate phosphatase (FBP) appear to have potential C_4_-specific activities. Among them, FBA2 (Si026480m) shows BS-preferential expression and is likely required for CBB function. FBA and FBP show M-preferential expression and are putatively involved in downstream sugar metabolism. The automated workflow also identified two ortholog groups with putative roles in the photorespiratory pathway (Fig. 1B, C), a catalase (CAT2, Si035374m) and a hydroxypyruvate reductase (HPR, Si017480m).

### Novel C_4_ candidate genes

In addition to the genes mentioned above, many candidate genes that had not been previously considered as C_4_ related (Fig. 1B, C and Supplementary Table S3) were identified by this method. They include three ortholog groups implicated in leaf development. Ortholog group Si028928m encodes an ADP-ribosylation factor-GTPase activating protein. Disruptions in the closest homolog from *Arabidopsis thaliana* (AT5G13300, VASCULAR NETWORK DEFECTIVE 1, VAN1) result in leaf vein patterning defects (Sieburth, 2006). Ortholog group SCL (Si026111m) is a GRAS family transcription factor and a homolog to SCARECROW-like 14 in *A. thaliana*. SCARECROW-like genes are known to be involved in endodermis pattern specification in roots in *A. thaliana*, and recently have been suspected of playing a key role in vasculature/BS/M patterning in leaves of C_4_ plants (Slewinski *et al.*, 2012). Another ortholog group with a potential link to leaf development is DRP5B (Si009435m), a dynamin-related family protein homologous to *A. thaliana* DRP5B, which is known to be involved in chloroplast division and development (Pyke and Leech, 1994).

Several potential C_4_-related transcription factors were also identified. Among them, a zinc finger homeodomain transcription factor (HB22, Si032496m) is of particular interest. It is homologous to a homeodomain transcription factor that has been shown to bind the promoter region of PEPC in dicot C_4_*Flaveria* species, but not to bind the promoter region of PEPC in C_3_*Flaveria* species (Windhövel *et al.*, 2001). The previously discussed SCL ortholog group (Si026111m) is also a transcription factor.

A gene ontology enrichment analysis using the GO annotations of homologous genes in *A. thaliana* showed a significant enrichment in molecular functions related to transporter activities (GO:0005215, FDR<0.05; Supplementary Table S5) among the 88 orthologous groups identified. In addition to the C_4_-related transporters described above, at least 12 other ortholog groups in our candidate list have predicted transporter functions. One of them is a putative sugar transporter (STP1, Si035219m), which shows preferential BS expression in both maize and *Setaria*. Many ortholog groups in the candidate list have never been linked to C_4_ photosynthesis, but some showed high significance in certain tests as well as BS/M differential expression profiles in maize and *Setaria* (Supplementary Table S3). One example is a glutamate receptor-like (GLR, ortholog group Si005804m) protein. Its homolog in *A. thaliana*, GLR3.4, has recently been shown to affect lateral root primordium formation through Ca^2+^ signaling pathways (Vincill *et al.*, 2013). As root development modules have been implicated in driving BS/M differentiation in C_4_ grasses (Slewinski *et al.*, 2012), we speculate that this gene may also have been co-opted from lateral root development in vein patterning of C_4_ grass leaves.

## Discussion

### Overview of the cross-species selection scans

In this study we have developed a genome-wide (6784 ortholog groups) unbiased survey for signals of positive selection or relaxed negative selection to discover genes related to C_4_ photosynthesis in six grass species. We used a relaxed FDR of <0.2 to capture a broad list of C_4_ candidate genes and identified a list of 88 candidate genes that have likely been co-opted into a C_4_ differentiation process (Supplementary Table S3). To develop a test for enrichment of C_4_-related genes identified in the selection scan, we compared the frequencies of known ‘C_4_ genes’ (carbon shuttle enzymes and transporters) in the set of 88 prioritized candidates with the total tested 6784 genes. Seven of the 11 known C_4_ genes were detected in the automated workflow. Thus, a significant enrichment in C_4_ genes was achieved using the automated workflow (Fisher’s exact test, *P*= 2.3×10^–9^). There are three major advantages of this evolutionary based approach for gene discovery. First, it does not require any *a priori* knowledge of C_4_ biochemistry or development to identify candidate genes, and is completely independent from expression and proteomics data (Huang and Brutnell, 2016). Second, it provides a much smaller list of candidate genes, defined by a robust statistical test, than other, ‘guilt by association’ techniques such as cell-type specific expression analysis and coexpression network clusters (Li *et al.*, 2010; Wang *et al.*, 2014*a*). Third, the automated nature of this cross-species selection scan workflow is also quite flexible. It may be expanded with new genomes/transcriptomes, and adopted for other traits under strong adaptive evolution in taxa of interest.

An important validation of this approach was revealed in the identification of known C_4_-associated genes including PEPC, PPDK, NADP-ME and OMT. However, as with most computationally based gene discovery platforms, the workflow suffers from both type I and type II errors. False positives can be caused by genes under selection due to other causes, relaxed negative selection rather than positive selection (e.g. pseudogenes in C_4_ lineages), or random fluctuations of *d*_N_/*d*_S_ (Yang, 2007). In the long run, these problems can be largely overcome through increasing species sampling, especially through increasing the number of phylogenetically independent C_3_–C_4_ comparisons (Christin *et al.*, 2007, 2009). This approach is feasible for grasses in particular, because C_4_ has originated in grasses at least 25 times (Grass Phylogeny Working Group II, 2012). New draft genomes/transcriptomes also provide more robust phylogenies for the tests performed and increase the specificity of detecting C_4_-related genes. More independent C_4_ lineages can also help with identifying genes under lineage-specific positive selection.

False negatives will not be as easily resolved through the inclusion of data from additional species. In addition to a large number of genes not recovered in synteny analysis, many ortholog groups are not considered due to complicated duplication/loss and mis-annotation, failing the multiple sequences alignment threshold, and/or failing the phylogeny congruence test (10 148 out of 16 934, ~59.9%), as a necessary sacrifice to ensure conservative predictions and automation of the workflow. As shown here, our false negatives included one core C_4_ carbon shuttle enzyme (CA) and four putative C_4_-related transporters. A key to solve this problem is to improve annotations of all genomes. It greatly reduces false gene losses (when a syntenic ortholog exists in one species but is not annotated), improves the quality of multispecies alignments and increases the chance of reconstructing the correct gene phylogeny. For example, probable candidate ortholog groups that are significant in manual tests could have been included in the automated workflow (e.g. BASS2 and PPT1; Supplementary Table S2) with improved genome annotations and/or alignments. Gene orthology calls based on gene synteny, if applied across a broader range of species, would improve existing gene annotations (Schnable *et al.*, 2012). Additionally, topology-based congruence tests for orthology may be substituted by a Bayesian statistical framework to test if an alignment-based gene tree significantly deviates from the expected (genome wide estimated) species tree to allow some more flexibility accounting for errors introduced by a small species sample and short alignments.

It is also likely that the protein sequences of some genes co-opted into C_4_ photosynthesis are simply not subject to positive selection. This could include proteins involved in non-rate limiting steps of metabolic networks (NADP-MDH is a potential example), or genes where adaption to a role in C_4_ photosynthesis occurs through mechanisms other than amino acid substitutions (e.g. copy number variation and/or *cis*-element-induced expression level changes). Accordingly, the method presented here is not comprehensive in identifying all C_4_-related genes in a group of species, but it does represent a novel and complementary approach to gene discovery based on biochemical or transcriptional characterizations.

### The *Setaria–Dichanthelium* clade is a key for C_4_ gene discoveries in grasses

Two additional phylogenetic conditions (condition 8, phylogeny without *Dichanthelium*, and condition 9, phylogeny without the *Setaria*–*Dichanthelium* clade; Fig. 1A and Supplementary Table S3) were used to determine the importance of the *Setaria*–*Dichanthelium* clade for our results. Clearly, the power to detect C_4_-related genes dramatically decreases under these two conditions (Fig. 1B, C). None of the three core C_4_ genes (PEPC, NADP-ME and PPDK) shows statistical significance at the FDR<0.2 level. Excluding the *Dichanthelium* branch alone is slightly better than excluding the entire *Setaria*–*Dichanthelium* clade, under which the detection power is lost almost completely. The lack of detection power is most likely due to the small number of sampled species and long divergence time between the panicoid and pooid lineages.

This result clearly shows the inclusion of the *Setaria*–*Dichanthelium* clade, a recently diverged C_3_–C_4_ species pair, is crucial for identifying C_4_-related genes using our approach. In the absence of such closely related C_3_–C_4_ pairs, it is often necessary to employ simple pairwise comparisons, frequently between long-diverged lineages such as rice vs. maize (Wang *et al.*, 2014*a*). This more recent C_3_–C_4_ comparison affords a dramatic increase of power in detecting signals of selection, suggesting that other methods such as expression profiling and proteomics could benefit from such comparisons as well. It also indicates that the inclusion of additional recently diverged C_3_–C_4_ comparisons will increase both the power and the specificity in revealing novelties associated with C_4_ gene evolution.

### Adaptive evolution in C_4_-related genes and its implications for engineering

As discussed above, signals of elevated *d*_N_/*d*_S_ were observed in many carbon shuttle enzymes and key transporters (Fig. 1), indicating changes in protein function that act to increase metabolic flux within the C_4_ cycle. These findings suggest that movement of metabolites between BS and M cells are potential rate limiting steps in C_4_ metabolism networks, consistent with prior metabolic modeling studies (Pick *et al.*, 2011; Wang *et al.*, 2014*b*). When considering the engineering of C_4_ photosynthesis into C_3_ plants, our findings point to ‘lessons learned’ from the evolutionary trajectories of C_4_ plants and reveal which enzymes and transporters may be necessary for insertion into C_3_ plants (Heckmann *et al.*, 2013; Wang *et al.*, 2014*b*). One example of such a component is the putative triose phosphate transporter (TPT), which is responsible for plastidial membrane transport of triose phosphate and 3-phosphoglycerate. While little engineering attention has been paid to this gene relative to core C_4_ genes such as PEPC and NADP-ME, recent modeling work has shown that the TPT is a critical component for the efficiency of C_4_ photosynthesis (Wang *et al.*, 2014*b*). Our findings support the conclusion that TPT is a good target for engineering. Furthermore, as TPT is functional in both BS and M, it is unlikely to be detected from BS/M differential expression analysis without *a priori* knowledge of the biochemistry (John *et al.*, 2014).

Another important finding with potential engineering significance is that while some C_4_ core enzymes are recruited in parallel, others are differentially recruited in different lineages. Such parallelism versus divergence is evident when considering the three C_4_ subtypes, which are named after the primary decarboxylases expressed in BS cells (Furbank, 2011). This might indicate an evolutionary trajectory in which the shared genes are more constrained in enzymatic activities (e.g. PEPC and PPDK), whereas decarboxylase recruitment was more flexible. In maize, for instance it appears that both NADP-ME and PEPCK pathways are both utilized (Wingler *et al.*, 1999; Pick *et al.*, 2011). The divergence in C_4_ transporters creates fascinating opportunities for cross-species engineering. One example is the NHD-BASS2 system in *Setaria*. Early physiological work indicated two types of M plastidial pyruvate uptake systems in C_4_ species: the maize–sorghum clade uses an H^+^-dependent pyruvate transport system, while *Setaria*, *Panicum* and many other non-Andropogoneae species rely on a Na^+^-dependent pyruvate transport system (Aoki *et al.*, 1992). In the C_4_ eudicot *Flaveria*, the homologous NHD-BASS2 system has been suggested to be responsible for pyruvate uptake in a Na^+^-dependent fashion in M cells (Furumoto *et al.*, 2011). We find that both NHD (Si029362m, automated workflow) and BASS2 (Si001591m, manual) orthologs are likely under strong selection pressure in *Setaria*, but not in maize and/or sorghum (Fig. 1; Supplementary Tables S2 and S3). In addition, both NHD and BASS2 are highly expressed in M of *Setaria* but not in maize (Chang *et al.*, 2012; John *et al.*, 2014). The combined results strongly indicate NHD-BASS2 is a *Setaria*-specific pyruvate transport system that is not operational in maize. Accordingly, insertion of the NHD-BASS2 complex into maize could facilitate pyruvate flux into M, and ultimately increase overall photosynthetic assimilation efficiency.

## Conclusions

C_4_ photosynthesis drives productivity in some of the most ecologically and agronomically important species on the planet, but a genetic dissection of C_4_ has been limited by the lack of resolution of available tools. Here we demonstrated the potential of cross-species selection scans, based on the concept of adaptive molecular evolution, as a powerful new method to identify candidate genes for C_4_ photosynthesis. Unlike current -omics based approaches for gene discovery, our method is independent of *a priori* knowledge of C_4_ biochemistry and results in a small list of candidate genes. Using this method, we have identified 88 candidate C_4_-related genes, including both known and novel genes. These candidates, along with the method, provide new insight into engineering plants with better photosynthetic efficiency, and engineering C_4_ photosynthesis into C_3_ plants. This approach can also be broadly applied to other traits under adaptive evolution and represents a powerful new approach to gene discovery.

## Supplementary data

Supplementary data are available at *JXB* online.

Table S1. Phylogenies used for positive selection test given maize duplication, gene loss in rice or *Brachypodium*, under different phylogenetic conditions.

Table S2. Manually conducted tests.

Table S3. Candidates from automated workflow.

Table S4. Gene names and syntenic ortholog group correspondence for six grass species.

Table S5. Gene ontology enrichment analysis using *Arabidospsis thaliana* homologs.

## Supplementary Material

Supplementary Table S1 and S4-S5

Supplementary Table S2

Supplementary Table S3

## References

[CIT0001] Aoki N Ohnishi J Kanai R 1992 Two different mechanisms for transport of pyruvate into mesophyll chloroplasts of C_4_ plants—a comparative study. Plant and cell Physiology 33, 805–809.

[CIT0002] Bailey KJ Battistelli A Dever LV Lea PJ Leegood RC 2000 Control of C_4_ photosynthesis: effects of reduced activities of phosphoenolpyruvate carboxylase on CO_2_ assimilation in *Amaranthus edulis* L. Journal of Experimental Botany 51, 339–346. 10938841 10.1093/jexbot/51.suppl_1.339

[CIT0003] Bennetzen JL Schmutz J Wang H 2012 Reference genome sequence of the model plant *Setaria*. Nature Biotechnology 30, 555–561. 10.1038/nbt.2196 22580951

[CIT0004] Brown WV 1975 Variations in anatomy, associations, and origins of Kranz tissue. American Journal of Botany 62, 395–402.

[CIT0005] Camacho C Coulouris G Avagyan V Ma N Papadopoulos J Bealer K Madden TL 2009 BLAST+: architecture and applications. BMC Bioinformatics 10, 421. 20003500 10.1186/1471-2105-10-421 PMC2803857

[CIT0006] Castresana J 2000 Selection of conserved blocks from multiple alignments for their use in phylogenetic analysis. Molecular Biology and Evolution 17, 540–552. 10742046 10.1093/oxfordjournals.molbev.a026334

[CIT0007] Chang Y-M Liu W-Y Shih AC-C 2012 Characterizing regulatory and functional differentiation between maize mesophyll and bundle sheath cells by transcriptomic analysis. Plant Physiology 160, 165–177. 22829318 10.1104/pp.112.203810 PMC3440195

[CIT0008] Christin P-A Boxall SF Gregory R Edwards EJ Hartwell J Osborne CP 2013 Parallel recruitment of multiple genes into C_4_ photosynthesis. Genome Biology and Evolution 5, 2174–2187. 24179135 10.1093/gbe/evt168 PMC3845648

[CIT0009] Christin P-A Petitpierre B Salamin N Buchi L Besnard G 2009 Evolution of C_4_ phosphoenolpyruvate carboxykinase in grasses, from genotype to phenotype. Molecular Biology and Evolution 26, 357–365. 18988688 10.1093/molbev/msn255

[CIT0010] Christin P-A Salamin N Savolainen V Duvall MR Besnard G 2007 C_4_ photosynthesis evolved in grasses via parallel adaptive genetic changes. Current Biology 17, 1241–1247. 17614282 10.1016/j.cub.2007.06.036

[CIT0011] Christin PA Samaritani E Petitpierre B Salamin N Besnard G 2010 Evolutionary insights on C_4_ photosynthetic subtypes in grasses from genomics and phylogenetics. Genome Biology and Evolution 1, 221–230. 10.1093/gbe/evp020 PMC2817415 20333192

[CIT0012] Christin P-A Spriggs E Osborne CP Stromberg CAE Salamin N Edwards EJ 2014 Molecular dating, evolutionary rates, and the age of the grasses. Systematic Biology 63, 153–165. 24287097 10.1093/sysbio/syt072

[CIT0013] Cousins AB Badger MR von Caemmerer S 2006 Carbonic anhydrase and its influence on carbon isotope discrimination during C_4_ photosynthesis. Insights from antisense RNA in *Flaveria bidentis*. Plant Physiology 141, 232–242. 16543411 10.1104/pp.106.077776 PMC1459309

[CIT0014] Du Z Zhou X Ling Y Zhang Z Su Z 2010 agriGO: a GO analysis toolkit for the agricultural community. Nucleic Acids Research 38, W64–W70. 20435677 10.1093/nar/gkq310 PMC2896167

[CIT0015] Edwards EJ Smith SA 2010 Phylogenetic analyses reveal the shady history of C_4_ grasses. Proceedings of the National Academy of Sciences of the United States of America 107, 2532–2537. 20142480 10.1073/pnas.0909672107 PMC2823882

[CIT0016] Furbank RT 2011 Evolution of the C_4_ photosynthetic mechanism: are there really three C_4_ acid decarboxylation types? Journal of Experimental Botany 62, 3103–3108. 21511901 10.1093/jxb/err080

[CIT0017] Furumoto T Yamaguchi T Ohshima-Ichie Y 2011 A plastidial sodium-dependent pyruvate transporter. Nature 476, 472–475. 21866161 10.1038/nature10250

[CIT0018] Giussani LM Cota-Sánchez JH Zuloaga FO Kellogg EA 2001 A molecular phylogeny of the grass subfamily Panicoideae (Poaceae) shows multiple origins of C_4_ photosynthesis. American Journal of Botany 88, 1993–2012. 21669633

[CIT0019] Grass Phylogeny Working Group II 2012 New grass phylogeny resolves deep evolutionary relationships and discovers C_4_ origins. New Phytologist 193, 304–312. 22115274 10.1111/j.1469-8137.2011.03972.x

[CIT0020] Heckmann D Schulze S Denton A Gowik U Westhoff P Weber AP Lercher MJ 2013 Predicting C_4_ photosynthesis evolution: modular, individually adaptive steps on a Mount Fuji fitness landscape. Cell 153, 1579–1588. 23791184 10.1016/j.cell.2013.04.058

[CIT0021] Huang P Brutnell TP 2016 A synthesis of transcriptomic surveys to dissect the genetic basis of C_4_ photosynthesis. Current Opinion in Plant Biology 31, 91–99. 27078208 10.1016/j.pbi.2016.03.014

[CIT0022] John CR Smith-Unna RD Woodfield H Covshoff S Hibberd JM 2014 Evolutionary convergence of cell-specific gene expression in independent lineages of C_4_ grasses. Plant Physiology 165, 62–75. 24676859 10.1104/pp.114.238667 PMC4012605

[CIT0023] Kinoshita H Nagasaki J Yoshikawa N Yamamoto A Takito S Kawasaki M Sugiyama T Miyake H Weber APM Taniguchi M 2011 The chloroplastic 2-oxoglutarate/malate transporter has dual function as the malate valve and in carbon/nitrogen metabolism: OMT in malate valve and C/N interaction. The Plant Journal 65, 15–26. 21175886 10.1111/j.1365-313X.2010.04397.x

[CIT0024] Kumar S Stecher G Peterson D Tamura K 2012 MEGA-CC: computing core of molecular evolutionary genetics analysis program for automated and iterative data analysis. Bioinformatics 28, 2685–2686. 22923298 10.1093/bioinformatics/bts507 PMC3467750

[CIT0025] Li P Ponnala L Gandotra N 2010 The developmental dynamics of the maize leaf transcriptome. Nature Genetics 42, 1060–1067. 21037569 10.1038/ng.703

[CIT0026] Mailund T Pedersen CNS 2004 QDist—quartet distance between evolutionary trees. Bioinformatics 20, 1636–1637. 14962942 10.1093/bioinformatics/bth097

[CIT0027] Majeran W van Wijk KJ 2009 Cell-type-specific differentiation of chloroplasts in C_4_ plants. Trends in Plant Science 14, 100–109. 19162526 10.1016/j.tplants.2008.11.006

[CIT0028] Ouyang S Zhu W Hamilton J 2007 The TIGR rice genome annotation resource: improvements and new features. Nucleic Acids Research 35, D883–D887. 17145706 10.1093/nar/gkl976 PMC1751532

[CIT0029] Paterson AH Bowers JE Bruggmann R 2009 The *Sorghum bicolor* genome and the diversification of grasses. Nature 457, 551–556. 19189423 10.1038/nature07723

[CIT0030] Pick TR Bräutigam A Schlüter U 2011 Systems analysis of a maize leaf developmental gradient redefines the current C_4_ model and provides candidates for regulation. The Plant Cell 23, 4208–4220. 22186372 10.1105/tpc.111.090324 PMC3269860

[CIT0031] Pyke KA Leech RM 1994 A genetic analysis of chloroplast division and expansion in *Arabidopsis thaliana*. Plant Physiology 104, 201–207. 12232072 10.1104/pp.104.1.201 PMC159178

[CIT0032] Sage RF 2004 The evolution of C_4_ photosynthesis. New Phytologist 161, 341–370. 10.1111/j.1469-8137.2004.00974.x 33873498

[CIT0033] Sage RF Christin P-A Edwards EJ 2011 The C_4_ plant lineages of planet Earth. Journal of Experimental Botany 62, 3155–3169. 21414957 10.1093/jxb/err048

[CIT0034] Sage RF Sage TL Kocacinar F 2012 Photorespiration and the evolution of C_4_ photosynthesis. Annual Review of Plant Biology 63, 19–47. 10.1146/annurev-arplant-042811-105511 22404472

[CIT0035] Schnable JC Freeling M Lyons E 2012 Genome-wide analysis of syntenic gene deletion in the grasses. Genome Biology and Evolution 4, 265–277. 22275519 10.1093/gbe/evs009 PMC3318446

[CIT0036] Schnable PS Ware D Fulton RS 2009 The B73 maize genome: complexity, diversity, and dynamics. Science 326, 1112–1115. 19965430 10.1126/science.1178534

[CIT0037] Sieburth LE 2006 SCARFACE Encodes an ARF-GAP that is required for normal auxin efflux and vein patterning in *Arabidopsis*. The Plant Cell 18, 1396–1411. 16698946 10.1105/tpc.105.039008 PMC1475492

[CIT0038] Slewinski TL Anderson AA Zhang C Turgeon R 2012 Scarecrow plays a role in establishing kranz anatomy in maize leaves. Plant and Cell Physiology 53, 2030–2037. 23128603 10.1093/pcp/pcs147

[CIT0039] Stamatakis A 2014 RAxML version 8: a tool for phylogenetic analysis and post-analysis of large phylogenies. Bioinformatics 30, 1312–1313. 24451623 10.1093/bioinformatics/btu033 PMC3998144

[CIT0040] Still CJ Berry JA Collatz GJ DeFries RS 2003 Global distribution of C_3_ and C_4_ vegetation: Carbon cycle implications. Global Biogeochemical Cycles 17, 6-1–6-14.

[CIT0041] Strimmer K 2008 fdrtool: a versatile R package for estimating local and tail area-based false discovery rates. Bioinformatics 24, 1461–1462. 18441000 10.1093/bioinformatics/btn209

[CIT0042] Studer AJ Gandin A Kolbe AR Wang L Cousins AB Brutnell TP 2014 A limited role for carbonic anhydrase in C_4_ photosynthesis as revealed by a *ca1ca2* double mutant in maize. Plant Physiology 165, 608–617. 24706552 10.1104/pp.114.237602 PMC4044840

[CIT0043] Szalkowski AM 2012 Fast and robust multiple sequence alignment with phylogeny-aware gap placement. BMC Bioinformatics 13, 129. 22694311 10.1186/1471-2105-13-129 PMC3495709

[CIT0044] Vicentini A Barber JC Aliscioni SS Giussani LM Kellogg EA 2008 The age of the grasses and clusters of origins of C_4_ photosynthesis. Global Change Biology 14, 2963–2977.

[CIT0045] Vincill ED Clarin AE Molenda JN Spalding EP 2013 Interacting glutamate receptor-like proteins in phloem regulate lateral root initiation in *Arabidopsis*. The Plant Cell 25, 1304–1313. 23590882 10.1105/tpc.113.110668 PMC3663269

[CIT0046] Vogel JP Garvin DF Mockler TC 2010 Genome sequencing and analysis of the model grass *Brachypodium distachyon*. Nature 463, 763–768. 20148030 10.1038/nature08747

[CIT0047] Wang L Czedik-Eysenberg A Mertz RA 2014 *a* Comparative analyses of C_4_ and C_3_ photosynthesis in developing leaves of maize and rice. Nature Biotechnology 32, 1158–1165. 10.1038/nbt.3019 25306245

[CIT0048] Wang X Gowik U Tang H Bowers JE Westhoff P Paterson AH 2009 Comparative genomic analysis of C_4_ photosynthetic pathway evolution in grasses. Genome Biology 10, R68. 19549309 10.1186/gb-2009-10-6-r68 PMC2718502

[CIT0049] Wang Y Long SP Zhu X-G 2014 *b* Elements required for an efficient NADP-malic enzyme type C_4_ photosynthesis. Plant Physiology 164, 2231–2246. 24521879 10.1104/pp.113.230284 PMC3982775

[CIT0050] Windhövel A Hein I Dabrowa R Stockhaus J 2001 Characterization of a novel class of plant homeodomain proteins that bind to the C_4_ phosphoenolpyruvate carboxylase gene of *Flaveria trinervia*. Plant Molecular Biology 45, 201–214. 11289511 10.1023/a:1006450005648

[CIT0051] Wingler A Walker RP Chen Z-H Leegood RC 1999 Phosphoenolpyruvate carboxykinase is involved in the decarboxylation of aspartate in the bundle sheath of maize. Plant Physiology 120, 539–546. 10364405 10.1104/pp.120.2.539 PMC59292

[CIT0052] Yang Z 2007 PAML 4: phylogenetic analysis by maximum likelihood. Molecular Biology and Evolution 24, 1586–1591. 17483113 10.1093/molbev/msm088

