# Supplementary material for: Cross species selection scans identify components of C4 photosynthesis in the grasses
Source: J Exp Bot. 2016 Jul 19;68(2):127–35. doi: 10.1093/jxb/erw256 (PMC5429014; doi:10.1093/jxb/erw256)
Supplement: Supplementary Table S1 and S4-S5 [file erw256_suppl_Supplementary_Table_S1_S4_S5.pdf]

Table S1. Phylogenies used for positive selection test given maize duplication, gene loss in rice or Brachypodium, under different phylogenetic conditions.

Duplication/Loss	Full[a] ❶	Exclude Zm[b] ❷	Exclude Sb ❸	Exclude Si ❹
Full[c]	(((Zm, Sb) #1, (Si #1, Do)), (Os, Bd))	((Sb #1, (Si #1, Do)), (Os, Bd))	((Zm #1, (Si #1, Do)), (Os, Bd))	(((Zm, Sb) #1, Do), (Os, Bd))
Duplication in Zm	((((Zm1, Zm2), Sb) #1, (Si #1, Do)), (Os, Bd))	((Sb #1,(Si #1, Do)), (Os, Bd))	(((Zm1, Zm2) #1, (Si #1, Do)), (Os, Bd))	((((Zm1, Zm2), Sb) #1, Do), (Os, Bd))
Loss in Os	(((Zm, Sb) #1, (Si #1, Do)), Bd)	((Sb #1, (Si #1, Do)), Bd)	((Zm #1, (Si #1, Do)), Bd)	(((Zm, Sb) #1, Do), Bd)
Loss in Bd	(((Zm, Sb) #1, (Si #1, Do)), Os)	((Sb #1, (Si #1, Do)), Os)	((Zm #1, (Si #1, Do)), Os)	(((Zm, Sb) #1, Do), Os)

Duplication/Loss	Exclude Zm-Sb ❺	Exclude Si-Sb ❻	Exclude Si-Zm ❼
Full[c]	((Si #1, Do), (Os, Bd))	((Zm #1, Do), (Os, Bd))	((Sb #1, Do), (Os, Bd))
Duplication in Zm	((Si #1, Do), (Os, Bd))	(((Zm1, Zm2) #1, Do), (Os, Bd))	((Sb #1, Do), (Os, Bd))
Loss in Os	Not considered[d]	Not considered	Not considered
Loss in Bd	Not considered	Not considered	Not considered

Duplication/Loss	Exclude Do ❽	Exclude Do-Si clade ❾
Full[c]	(((Zm, Sb) #1, Si #1), (Os, Bd))	((Zm, Sb) #1, (Os, Bd))
Duplication in Zm	((((Zm1, Zm2), Sb) #1, Si #1), (Os, Bd))	(((Zm1, Zm2), Sb) #1, (Os, Bd))
Loss in Os	(((Zm, Sb) #1, Si #1), Bd)	Not considered
Loss in Bd	(((Zm, Sb) #1, Si #1), Os)	Not considered

[a] Zm: maize; Sb: sorghum; Si: *Setaria italia*; Do: Dichanthelium; Bd: *Brachypodium distachyon*; Os: rice

[b] The "#1" sign designate C_4 leading branches on the testing phylogeny

[c] The full phylogenies are shown in Fig. 1

[d] Situation not considered due to only 3 taxa left

Table S4. Gene names and syntenic ortholog group correspondence for six grass species

Gene name	Ortholog group name (*Setaria*)	maize	sorghum	rice	*Brachypodium*	*Dichanthelium*[a]
**NADP-ME**	Si000645m	GRMZM2G085019	Sobic.003G036200	LOC_Os01g09320	Bradi2g05620	Do024386.1
**PPDK**	Si021174m	GRMZM2G306345, GRMZM2G097457	Sobic.009G132900	LOC_Os05g33570	Bradi2g25745	Do015534.1
**PEPC**	Si005789m	GRMZM2G083841	Sobic.010G160700	NA[b]	Bradi1g39167	Do021545.1
**CA**[c]	Si003882m	GRMZM2G121878 (fused protein)[d]	Sobic.003G234200	LOC_Os01g45274	Bradi2g44856	Do001290.1
**NADP-MDH**	Si013632m	GRMZM2G129513	Sobic.007G166200	LOC_Os08g44810	Bradi3g12460	Do003942.1
**PCK**	Si034404m	GRMZM5G870932, GRMZM2G001696	Sobic.001G432800	LOC_Os03g15050	Bradi1g67730	Do007037.1
**AspAT1**	Si017156m	GRMZM5G836910	Sobic.004G331700	LOC_Os02g55420	Bradi3g54200	Do004307.1
**AspAT2**	Si001361m	GRMZM2G094712	Sobic.003G303300	LOC_Os01g55540	Bradi2g50500	Do016694.1
**OMT**	Si024403m	GRMZM2G383088	Sobic.008G112300	LOC_Os12g33080	Bradi4g06300	Do017525.1
**DCT1**	Si029415m	GRMZM2G040933, GRMZM2G515874	Sobic.002G233700	LOC_Os09g29430	Bradi4g32730	Do013844.1
**DCT4**[c]	Si035016m	N.A.	Sobic.004G035500	N.A.	N.A.	N.A.
**DCT2**	Si013503m	GRMZM2G086258	Sobic.007G226800	LOC_Os08g37600	Bradi3g38580	Do011021.1
**MEP3_a**	Si024315m	GRMZM2G305851	Sobic.009G124000	LOC_Os05g32230	Bradi2g26950	Do020718.1
**MEP3_b**	Si000451m	GRMZM2G099036	Sobic.003G341300	LOC_Os01g61120	Bradi2g53780	Do007926.1
**MEP3_c**	Si005376m	GRMZM2G138258	Sobic.003G431900	LOC_Os01g72710	Bradi2g61270	N.A.
**NHD**	Si029362m	GRMZM2G053927	Sobic.002G141900	LOC_Os09g02214	Bradi4g08260	Do023727.1
**BASS2**[c]	Si001591m	N.A.	Sobic.003G236800	LOC_Os01g45750	Bradi2g45100	Do010383.1
**PPT1**	Si013874m	GRMZM2G174107	N.A.	N.A.	N.A.	Do023872.1
**PPT2**	Si005351m	GRMZM2G066413	Sobic.003G050800	LOC_Os01g07730	Bradi2g04447	Do025241.1
**TPT**	Si001693m	GRMZM2G070605	Sobic.003G002300	LOC_Os01g13770	Bradi2g08340	Do017690.1
**FBA3**	Si010312m	GRMZM2G046284	Sobic.008G053200	LOC_Os12g07210	NA	Do000279.1
**FBA2**	Si026480m	GRMZM2G155253	Sobic.005G056400	LOC_Os11g07020	Bradi4g24367	Do010362.1
**FBP**	Si002088m	GRMZM5G836250	Sobic.003G367500	LOC_Os01g64660	Bradi2g56030	Do003645.1
**FBA1**	Si006745m	GRMZM2G345493	Sobic.010G188300	LOC_Os06g40640	Bradi1g36260	Do000618.1
**CAT2**	Si035374m	GRMZM2G090568	Sobic.001G517700	LOC_Os03g03910	Bradi1g76330	Do015295.1
**HPR**	Si017480m	GRMZM2G074282	Sobic.004G001300	LOC_Os02g01150	Bradi3g00330	Do025649.1
**VAN1**	Si028928m	GRMZM2G059225	Sobic.002G260700	LOC_Os09g33600	Bradi4g34880	Do005223.1
**SCL**	Si026111m	GRMZM5G895672	Sobic.005G231000	N.A.	Bradi4g09160	Do021853.1
**DRP5B**	Si009435m	GRMZM2G005848	Sobic.008G058000	LOC_Os12g07880	Bradi4g41137	Do011342.1
**STP1**	Si035219m	GRMZM2G374812	Sobic.001G543500	LOC_Os03g01170	Bradi1g78400	Do022428.1
**HB22**	Si032496m	GRMZM2G389379	Sobic.002G231100	LOC_Os09g29130	Bradi4g32580	Do019488.1
**GLR**	Si005804m	GRMZM2G148807	Sobic.010G231300	LOC_Os06g46670	Bradi1g32800	Do001711.1

[a] All *Dichanthelium* orthologs are determined by BLASTN and gene phylogeny instead of gene synteny

[b] N.A. indicates no clear syntenic orthoglogs found in the corresponding species

[c] For these genes the homologs are manually determined using BLASTN, and orthology is verified by phylogeny and gene synteny when available.

[d] GRMZM2G121878 is known to be a fused protein of two tandem copies (Studer *et al*. 2014).

Table S5. Gene ontology enrichment analysis using Arabidospsis thaliana homologs

GO term	Ontology	Description	Number in input list	Number in BG/Ref	p-value	FDR
GO:0015672	P	monovalent inorganic cation transport	5	32	0.00017	0.042
GO:0015291	F	secondary active transmembrane transporter activity	8	65	1.40E-05	0.00097
GO:0022891	F	substrate-specific transmembrane transporter activity	12	192	0.00012	0.0045
GO:0015297	F	antiporter activity	5	33	0.0002	0.0048
GO:0022892	F	substrate-specific transporter activity	12	222	0.00046	0.0066
GO:0022857	F	transmembrane transporter activity	13	248	0.00037	0.0066
GO:0015075	F	ion transmembrane transporter activity	9	139	0.0006	0.0072
GO:0005215	F	transporter activity	14	317	0.0012	0.011
GO:0022804	F	active transmembrane transporter activity	9	152	0.0011	0.011
